# Hierarchically Nanoporous Pyropolymers Derived from Waste Pinecone as a Pseudocapacitive Electrode for Lithium Ion Hybrid Capacitors

**DOI:** 10.1038/s41598-020-62459-0

**Published:** 2020-04-02

**Authors:** Jong Chan Hyun, Jin Hwan Kwak, Sang Moon Lee, Jaewon Choi, Kyu-Tae Lee, Young Soo Yun

**Affiliations:** 1 Department of Chemical Engineering, Kangwon National University, Samcheok, 25913 South Korea; 2 KU-KIST Graduate School of Converging Science and Technology, Korea University, 145 Anam-ro, Seongbuk-gu, Seoul, 02841 Republic of Korea; 3 Research Center for Materials Analysis, Korea Basic Science Institute(KBSI), 169-148 Gwahak-ro, Yuseong-gu, Daejeon 34133 Korea; 4 Department of Chemistry and Research Institute of Natural Sciences, Gyeongsang National University, Jinju, 52828 Korea; 5 Department of Physics, Inha University, Incheon, 22212 South Korea

**Keywords:** Supercapacitors, Porous materials

## Abstract

The non-aqueous asymmetric lithium ion hybrid capacitor (LIHC) is a tactical energy storage device composed of a faradic and non-faradic electrode pair, which aims to achieve both high energy and great power densities. On the other hand, the different types of electrode combinations cause severe imbalances in energy and power capabilities, leading to poor electrochemical performance. Herein, waste pinecone-derived hierarchically porous pyropolymers (WP-HPPs) were fabricated as a surface-driven pseudocapacitive electrode, which has the advantages of both faradic and non-faradic electrodes. The unique materials properties of WP-HPPs possessing high effective surface areas and hierarchically open nanopores led to high specific capacities of ~412 mA h g^−1^ and considerable rate/cycling performance as a cathode for LIHCs. In particular, nanometer-scale pores, approximately 3 nm in size, plays a key role in the pseudocapacitive charge storage behaviors because open nanopores can transport solvated Li-ions easily into the inside of complex carbon structures and a large specific surface area can be provided by the effective active surface for charge storage. In addition, WP-HPP-based asymmetric LIHCs assembled with a pseudocapacitive counterpart demonstrated feasible electrochemical performance, such as maximum specific energy and specific power of ~340 Wh kg^−1^ and ~11,000 W kg^−1^, respectively, with significant cycling stability.

## Introduction

The rapid growth of modern technology has accelerated the use of diverse state-of-the-art electronic devices that require suitable power sources^1,2^. Accordingly, Li ion batteries (LIBs) are used in a wide variety of electronics because of their high energy density (~200 Wh kg^−1^), high round-trip energy efficiency, useful power capability (<1000 W kg^−1^), and long-term cycle life (<1,000 times)^3,4^. On the other hand, the broad range of application devices sometimes require a higher power density and longer lifespan, requiring a diversification of applicable power sources^5^. Supercapacitors can deliver a high power density (>10,000 W kg^−1^) and cycling stability of more than 10,000 charge discharge cycles, and they have a simple electrode configuration composed of the same electrode pair that includes inexpensive active electrode materials (AEMs)^6,7^. Therefore, supercapacitors are partly superior to LIBs, but LIBs have a large portion of the market owing to their higher energy densities. As one of the strategies to achieve a supercapacitor-like high power density and LIB-like high energy density, an asymmetric electrode configuration based on a mixed electrode combination is suggested as Li ion hybrid capacitors (LIHCs)^8–10^. When faradic and non-faradic AEMs are assembled as an electrode pair for LIHCs, the sluggish charge transport rate of faradic AEMs deteriorates the kinetics of non-faradic AEMs^11,12^. In addition, the relatively poor capacity of non-faradic AEMs cause significant energy loss in faradic AEMs^13,14^. The power and energy imbalance between the different types of AEMs exacerbate their respective disadvantages, leading to poor energy and power performance^11–15^. To address the energy and power imbalances, considerable research efforts have been reported, where several nanostructured AEMs were developed as both the anode and cathode^16–27^. From the results, the high-power anode can be achieved by nanoscale effects, whereas most of the reported carbon-based cathodes showed a specific capacity of only <150 mA h g^−1^, which is a much lower value than that of the anode counterpart (>400 mA h g^−1^)^23–26^. Nevertheless, it is difficult to find alternative AEMs because of the strong advantages of carbon-based materials, such as mass-scalability, simple chemistry, and well-established preparation process. Therefore, more studies should focus on carbon-based high-energy AEMs as a cathode for LIHCs.

Pyropolymer is a carbonaceous compound with high functionality and highly tunable properties, which can be prepared from natural polymer precursors, such as cellulose and proteins by a low temperature pyrolysis process^28–31^. According to the precursor and fabrication process, a wide range of material properties can be achieved, and mass-scalable and tractable characteristics of pyropolymers make them more powerful in several application fields. In particular, bio-abundant lignocellulose-based materials, such as waste pinecone (WP), can be a useful precursor material to prepare pyropolymer because of their rigid molecular structure and high intermolecular interaction. Recently, considerable research results for WP-derived pyropolymers (WP-PPs) for use as biofuel^32^, catalyst^33^, absorbent^34,35^, and energy storage have been reported^36,37^. These results show that WP-PPs have good material properties for a range of applications. On the other hand, there are no reports on the pseudocapacitive charge storage performance of WP-PPs as a cathode for LIHCs. In addition, the relationship between the pore structure and pseudocapacitive charge storage performance of the pyropolymers for Li ion storage has yet to be unveiled.

In this study, high-performance pseudocapacitive AEMs as a cathode for LIHCs were fabricated from WP using a simple thermal treatment method. The WP-derived hierarchically porous pyropolymers (WP-HPPs) have outstanding material properties, such as a high specific surface area of ~3411.2 m^2^ g^−1^ and multimodal nanoporous structure, including a large number of topological defects and chalcogen heteroatoms (C/O ratio of 10.4). In particular, open nanopores, a few nanometers in size, play a key role in the pseudocapacitive lithium ion storage performance in the cathodic voltage region of 1.0~4.5 V vs. Li^+^/Li. When a WP-HPP-based cathode was assembled with a pseudocapacitive pyropolymer anode called as nitrogen-rich pyropolymer nanofiber (N-PN)^30^, the pseudocapacitive electrode pair showed well-balanced electrochemical performance, achieving a significantly high specific energy of ~340 Wh kg^−1^ and a high specific power of ~11,000 W kg^−1^ with high cycling stability over two thousand galvanostatic charge/discharge cycles.

## Experimental Method

### Preparation of WP-HPPs

WPs collected around campus were washed several times with ethanol and distilled water. The purified WPs were dried in a convection oven at 80 °C for 12 h and treated thermally at 600 °C for 2 h in a tubular furnace under an Ar flow of 150 ml min^−1^. The carbonized WPs were ground in a mortar and mixed with potassium hydroxide at 200, 400, 600 or 800 wt. %, which were then heated in a tubular furnace at 800 °C for 2 h under an Ar flow of 150 ml min^−1^. The heating rate was 5 °C min^−1^. The resulting products, called 2-, 4-, 6-, and 8-WP-HPPs according to the potassium hydroxide to WPs weight ratio, were washed with distilled water and ethanol several times and stored in a vacuum oven at 30 °C with no further treatment.

### Characterization

The morphology and carbon microstructures of the WP-HPPs were analyzed by field emission scanning electron microscopy (FE-SEM, S-4300SE, Hitachi, Japan) and field emission transmission electron microscopy (FE-TEM, JEM2100F, JEOL, Japan). X-ray diffraction (XRD, Rigaku, DMAX 2500) was conducted using Cu Kα radiation (λ = 0.154 nm) at 40 kV and 100 mA with a range of 5–60° 2θ. The Raman spectra were measured using a continuous linearly polarized laser with a wavelength of 532 nm and a 1200 groove/mm grating. The chemical structure of the WP-HPPs was investigated by X-ray photoelectron spectroscopy (XPS, PHI 5700 ESCA, Chanhassen, USA) using monochromatic Al Kα radiation. The porosity of the samples was characterized by analysis of N_2_ adsorption –desorption isotherms at 77 K, CO_2_ adsorption at 273 K (ASAP2020, Micromeritics, USA). Before measurement, the samples were degassed below 1.33 × 10^3^ Pa for 1 hr and heated (5 K/min) to 393 K over-night. The microporous volume (V_mic_), and the external surface area (S_ext_) were determined by means of t-plot. The total pore volume (V_tot_) was calculated at relative pressure of P/P_0_ = 0.98. The pore size distributions (PSDs) were analyzed using the Barret–Joyner–Halenda (BJH) method, Density Functional Theory (DFT) method, Horvath-Kawazoe (H-K) method. On the other hand, ultra-micropore size distribution (UMPSD) of samples were determined by CO_2_ adsorption.

### Electrochemical characterization

The electrochemical performance of the WP-HPP cathode, N-PN anode^30^ and their full cells was characterized using a Wonatech automatic battery cycler and CR2032-type coin cells. For the half-cell tests, coin cells were assembled in a glovebox filled with argon using the samples as the working electrode and metallic Li foil as the reference and counter electrodes. LiPF_6_ (1 M; Sigma-Aldrich, 98%) was dissolved in a solution of ethylene carbonate (EC) and dimethyl carbonate (DMC) (1:1 v/v) and used as the electrolyte for both the half-cell and full cell tests. A glass microfiber filter (GF/F, Whatman) was used as a separator. The working electrodes were prepared by mixing the active material (90 wt. %) and polyvinylidene fluoride (10 wt. %) in N-methyl-2-pyrrolidone. The resulting slurry was applied uniformly to the Al or Cu foil. The electrodes were dried at 120 °C for 2 h and roll pressed. The average active material loading was ~1 mg cm^−2^. For a full cell test, the total electrode weight was ~2 mg, in which the same AEM weight contents were used. The respective anode and cathode were pre-cycled for 10 cycles as a half-cell configuration with Li metal, and they were re-assembled as a full cell. Electrochemical impedance spectroscopy (EIS) tests were performed at room temperature in the frequency range of 0.1 MHz to 50 mHz using an impedance analyzer (ZIVE SP1, WonATech).

## Results and Discussion

**Figure 1:**
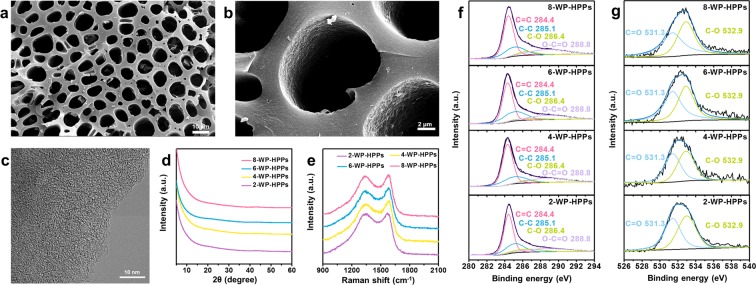
Materials properties of WP-HPPs. (**a**,**b**) FE-SEM images of 6-WP-HPPs at different magnifications and (**c**) FE-TEM image of 6-WP-HPPs. (**d**) XRD patterns, (**e**) Raman spectra and XPS (f) C 1s and (**g**) O 1s spectra of WP-HPPs.

Macroporous morphologies of WP-HPPs and pyropolymer precursors which are prepared by pyrolysis process of the purified WPs at 600 °C, are depicted in Figs. 1(a,b) and S1. Pores, a few micrometers in diameter with a random shape are spread over the overall area, and the high-magnification image shows the rough inner surface of the macropores [Fig. 1(b)]. All the samples exhibit similar shapes to each other, suggesting that they are hard carbon-type polymer precursors that maintain their original morphologies during a carbonization process [Figs. 1(a,b) and S1]. In the heating process, the cellulose-based macromolecular structures of WPs can be transformed to relatively thermo-stable carbonaceous materials, which are composed mainly of two-dimensional poly-hexagonal carbon (PHC) layers^38^. The newly formed PHC structure has a large number of defect sites that crumple the carbon layers into stereoscopic shapes, causing poor stacking ordering. In particular, potassium hydroxide strongly attacks the PHC materials produced, resulting in more defective carbon structures, including multitudinous intrinsic topological defects and extrinsic heteroatom defects. The surface topologies of the samples were characterized by high-resolution FE-SEM observation [Fig. S2]. 6-WP-HPPs have the roughest surface topology in all samples, which is closely related to their carbon microstructure and pore structure. High-resolution FE-TEM images of the WP-HPPs demonstrate their amorphous structure with no distinct graphitic ordering [Figs. 1(c) and S3]. All the WP-HPPs reveal highly disordered carbon structures with a poor graphic lattice regardless of the amount of potassium hydroxide added [Figs. 1(c) and S4]. Poor graphitic ordering is also observed by XRD [Fig. 1(d)], which shows no characteristic peak. In contrast, the Raman spectra of WP-HPPs represent two fused and broad peaks at 1338 and 1580 cm^−1^ [Fig. 1(e)]^39^. The peak pair is well-known as the *D* and *G* bands caused by the disordered A_1g_ breathing mode of the poly-hexagonal carbon structures and the E_2g_ vibration mode of the six-membered aromatic ring, repectively^40,41^. The broad peak pair is deconvoluted to confirm more specific *G* to *D* intensity ratio (I_G_/I_D_) [Fig. S5]. The I_G_/I_D_ value is ~0.9. The presence of the peak pair, which has a similar intensity ratio, indicates that the WP-HPPs are composed of a few nanometer-sized defective poly-aromatic ring structures^41^. A three-dimensional aggregation of the crumpled PHC building blocks with several nanometer-sized dimensions leads to a nanoporous structure, which can be tuned by controlling the degree of activation with chemical reagents. The pore structures of the WP-HPPs with activation agents are addressed in detail after the following section.

The extrinsic defects on the WP-HPPs were characterized by XPS [Fig. 1(f,g)]. In the deconvoluted C 1s spectra of all samples, several chemical structures, such as sp^3^ C–C bonding, C–O bonding, and O–C=O bonding, show intensive sp^2^ C=C bonding, suggesting the presence of many topological defects and hydrophilic functional groups on the poly aromatic carbon structures [Fig. 1(f)]. The deconvoluted O 1s spectra reveal that the oxygen groups consist of two major groups, such as C–O and C=O bonding structures [Fig. 1(g)]. An appropriate oxygen dopant can increase the wettability of WP-HPPs with a carbonate-based electrolyte and work as redox active site for charge storage. The C/O ratios of WP-HPPs are 11.8, 11.3, 10.4, and 10.3 for 2-, 4-, 6-, and 8-WP-HPPs, respectively. Although the oxygen contents on WP-HPPs increase with increasing concentration of activation agents, their C/O ratios are similar. Considering that the oxygen functional groups are mostly introduced on defective sites (particularly on edge-defects), a more defective structure is expected on WP-HPPs prepared with larger levels of potassium hydroxide. In addition, the significantly high oxygen to carbon ratio suggests that most of the carbon edge sites are functionalized by oxygen functional groups. According to previously reported results, the oxygen functional groups on nanostructured carbon-based materials can be a redox host for a pseudocapacitive Li-ion storage^42–44^. Therefore, WP-HPPs with a high oxygen content have great potential as an active electrode material for Li-ion storage.

**Figure 2:**
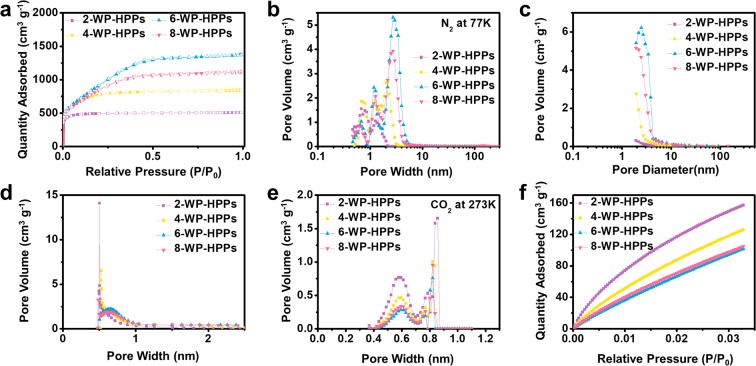
Porous properties of WP-HPPs. (**a**) nitrogen adsorption and desorption isotherm curves at 77 K, pore size distribution obtained from the nitrogen adsorption/desorption behaviors using the (**b**) DFT method (adsorption branch), (**c**) BJH method (desorption branch) and (**d)** HK method (adsorption branch), (**e**) pore size distribution obtained from carbon dioxide adsorption behaviors by the DFT method, and (**f**) carbon dioxide adsorption isotherms at 273 K.

Because the pseudocapacitive charge storage behaviors depend strongly on the active surface area, the porous properties of WP-HPPs were investigated using nitrogen adsorption and desorption isotherm tests [Fig. 2(a–d)]. The isotherm curves show a large amount of monolayer nitrogen adsorption and hysteresis-free shapes [Fig. 2(a)]. In particular, the isotherm curve of the 2-WP-HPPs shows a typical microporous structure corresponding to the Type–I of International Union of Pure and Applied Chemistry (IUPAC). The adsorbed nitrogen quantity increases dramatically with increasing potassium hydroxide concentration to 600 wt.%, and the external specific surface areas calculated from a t-plot are also increased more than 30 fold from 31.5 m^2^ g^−1^ to 1008.2 m^2^ g^−1^, indicating a sharp increase of a few-nanometer-sized pores (See Table 1). The PSD curves obtained using DFT show the differences in the pore structure of WP-HPPs more clearly [Fig. 2(b)]. The PSD curve of 2-WP-HPPs exhibits two distinctive peaks at 0.7 and 1.2 nm, whereas the curves of the other samples show an additional peak with a higher intensity at a larger pore width between 2~3 nm. Ultra-micropores (<0.7 nm) in a disordered carbon structure originate from misaligned stacking between the two distorted PHC layers, where the pore size could be dependent on the defective carbon structures [Fig. S5(a)]. In contrast, the micropores between 0.7~2 nm are induced from the aggregation of several distorted PHC layers in three-dimensional space, as shown in Fig. S5(b). Conventional activated carbon contains mainly ultra-micropores and micropores because they are complex three-dimensional assemblies composed of multitudinous defective PHC building blocks [Fig. S5(c)]. The PSD curve of 2-WP-HPPs shows they are a typical microporous carbon. On the other hand, the presence of the largest pores (2~3 nm in width) in 4-, 6- and 8-WP-HPPs is noteworthy. The selective etching of more defective PHC layers, which have very unstable carbon structures, produce the nanopores in the heating process with excessive potassium hydroxide. As a result, a larger pore rolled into one can form according to the removal of a carbon layer [Fig. S5(d)]. 6-WP-HPPs have the largest pore diameter of both ~1.2 nm and ~3 nm in all samples. The PSD data obtained from the BJH method also coincide with the results of the DFT method [Fig. 2(c)], wherein the highest nanopore volume of 6-WP-HPPs can be confirmed. On the other hand, the BJH method has a limitation for detecting small pores in a sub-nanometer scale. In the case of ultra-micropore characterization, information that is more detailed can be obtained using a carbon dioxide-based method, owing to the smaller kinetic diameter of carbon dioxide than that of nitrogen. The UMPSD of the WP-HPPs characterized by the nitrogen adsorption and desorption isotherm curve did not provide a significant difference for them [Fig. 2(d)], whereas the UMPSD results characterized by carbon dioxide at 273 K show clear information [Fig. 2(e)]. Interestingly, the UMPSD of the WP-HPPs obtained by the DFT method, as shown in Fig. 2(b), is in complete opposition to the result from carbon dioxide-based characterization. The 2-WP-HPPs have the largest ultra-micropore volume at ~0.58 nm, whereas the 6-WP-HPPs show the lowest ultra-micropore volume [Fig. 2(f)]. Accordingly, 2-WP-HPPs can store a much higher CO_2_ quantity with increasing relative pressure, even though their Brunauer-Emmett-Teller (BET) surface area (1917.1 m^2^ g^−1^) was only ~56% of that of the 6-WP-HPPs (3411.2 m^2^ g^−1^). These results suggest that the accommodation of guest molecules on WP-HPPs is strongly affected by PSD rather than the specific surface area. In addition, the results also suggest that the charge adsorption/desorption behaviors on WP-HPPs could be closely connected to their pore structure. Table 1 lists the detailed textural properties of WP-HPPs.

**Table 1:** Textural properties of WP-HPPs.

Sample	^a^S_BET_	^b^S_ext_	^c^V_mic_	^d^V_tot_
2-WP-HPPs	1,917.1	31.5	0.76	0.79
4-WP-HPPs	2,822.3	108.7	1.20	1.31
6-WP-HPPs	3,411.2	1,008.2	1.35	2.12
8-WP-HPPs	3,178.8	331.5	1.43	1.75

^a^BET surface area (m^2^ g^−1^).

^b^External surface area (m^2^ g^−1^).

^c^Micropore volume (m^2^ g^−1^).

^d^Total pore volume (cm^3^ g^−1^).

**Figure 3:**
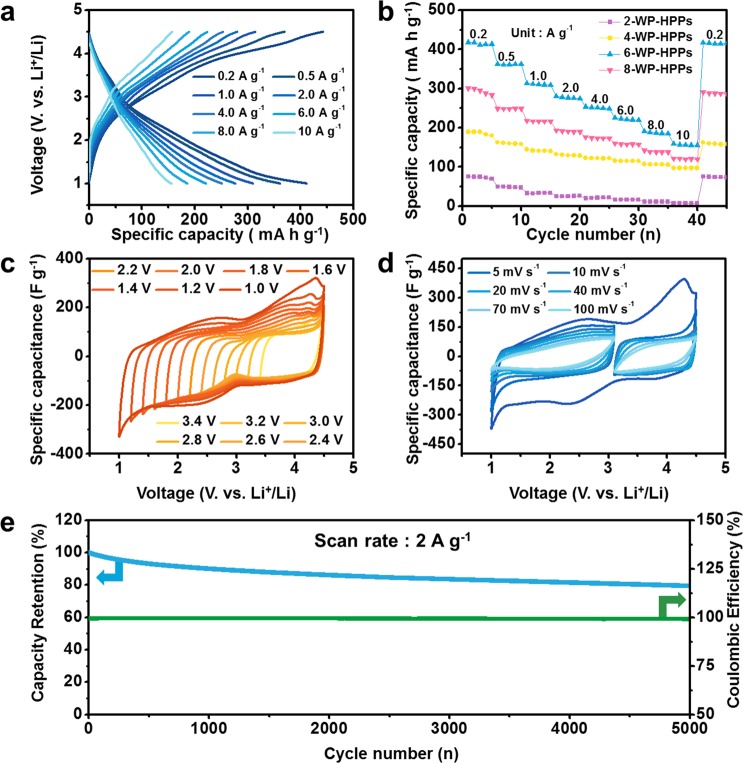
Electrochemical performance of 6-WP-HPPs in an electrolyte of 1 M LiPF_6_ dissolved in EC/DMC (1:1 v/v) over a voltage window of 1.0~4.5 V vs. Li^+^/Li. (**a**) galvanostatic charge/discharge profiles at different current rates, (**b**) rate capabilities of WP-HPPs at different current rates, (**c**) CV curves in different voltage ranges at a scan rate of 5 mV s^−1^, (**d**) CV curves at different scan rates from 5 to 100 mV s^−1^ in different voltage ranges, and (**e**) cycling performance at a current rate of 2 A g^−1^ during 5,000 cycles.

The practical charge storage performances of WP-HPPs with different pore structures were tested in an electrolyte of 1 M LiPF_6_ dissolved in an ethylene carbonate (EC) and dimethyl carbonate (DMC) mixture (1:1 v/v) solution over a voltage window of 1.0~4.5 V vs. Li^+^/Li [Fig. 3]. The stability of the EC/DMC mixture in the high cutoff voltage (4.5 V) was confirmed by a linear sweep voltammetry test from open circuit voltage to 4.5 V at a scan rate of 1 mV s^−1^ [Fig. S6]. The 6-WP-HPPs show a high specific discharge capacity of ~412 mA g^−1^ at 0.2 A g^−1^, which is a much higher value than them of the pyropolymer precursor (~18 mA h g^−1^) and the 2-, 4- and 8-WP-HPPs (~75, ~190 and ~295 mA h g^−1^, respectively) [Figs. 3(a) and S7]. In addition, the specific capacity of 6-WP-HPPs is higher than them of the previously reported carbon-based electrode materials [See Table S1]. The large differences in specific capacity could be due to their different pore structures because WP-HPPs have similar defect and surface properties but distinctively different pore structures. Under the organic electrolyte system, the solvated charges could have diameters several times larger than the bare charge (lithium ion), limiting the accessibility of lithium ions into smaller pores, such as ultra-micropores. This means that most of the specific surface areas in the 2-WP-HPPs are unusable because of the very narrow pore size. In contrast, the hierarchically nanoporous structure of 6-WP-HPPs can deliver charges more easily to the overall internal surfaces under a given kinetic condition. In addition, the better charge affinity originating from the hydrophilic properties can increase effective surface area with redox-active functional groups, which can store charges by nanoconfinement effects^45^ and super-ionic behaviors^46^. Therefore, 6-WP-HPPs reveal high specific capacities and great rate capabilities in the current range from 0.2 to 10 A g^−1^. Approximately 155 mA h g^−1^ is maintained at 10 A g^−1^ despite the gradual decrease in specific capacity with increasing current density [Fig. 3(b)]. The high rate capability of 6-WP-HPPs was confirmed by electrochemical impedance spectroscopy (EIS) analysis [Fig. S8]. The EIS profiles of WP-HPP samples show the similar shapes with one semicircle corresponding to charge transfer resistance (R_ct_) in the high frequency section, while the size of the semicircles is different in the respective samples [Fig. S8]. The R_ct_ value is decreased with increasing pore size, and 6-WP-HPPs and 8-WP-HPPs show the similar R_ct_ values because they have the similar pore size distribution (PSD), as shown in Fig. 2(b). These results indicate that the pore size plays a key role in charge storage kinetics of WP-HPP samples and the hierarchical pore structure of 6-WP-HPPs is highly effective to reduce the R_ct_ value^47,48^.

To understand the reason for the high-performance, the specific origin of the charge storage mechanism on 6-WP-HPPs was characterized further by voltage-controlled cyclic voltammetry (CV) [Fig. 3(c,d)]. In a limited voltage range between 3.4~4.5 V vs. Li^+^/Li, the CV curve shows a rectangular-like shape exhibiting capacitive behavior with a relatively smaller specific capacitance [Fig. 3(c)]. With increasing voltage range from 4.5 to 3.2, 3.0, 2.8, and 2.6 V, the CV curves change slightly and their specific capacitances are similar to the initial one. On the other hand, further increases in the voltage ranges cause a significant increase in specific capacitance. The capacitance increases with increasing voltage range, indicating the presence of pseudocapacitance because the specific capacitance induced from physical charge adsorption/desorption behaviors is basically a voltage-independent value. In particular, the specific capacitance is enhanced remarkably, when the voltage range is extended to the lower voltage area, where lithium ions act as a charge carrier [Fig. 3(c)]. Several studies showed that oxygen functional groups of carbon-based cathode materials can be a redox host for lithium ion storage at a cathodic voltage range^42–44^, and the CV profiles of 6-WP-HPPs exhibit a highly voltage-dependent CV curve. Therefore, the gradual capacitance growth on the oxygen-rich 6-WP-HPPs could be attributed to pseudocapacitive lithium ion storage. The pseudocapacitive lithium ion storage ratio in overall capacitance can be calculated by using the followed equation, *i*(*V*) = *k*_1_*v* + *k*_2_*v*^1/2^, where *ν* is the sweep rate^49–51^. The pseudocapacitance ratio of 6-WP-HPPs is calculated as ~35% [Fig. S9]. Figure 3(d) shows the voltage-dependent charge storage behaviors more clearly. The cyclovoltammograms depicts them of symmetric capacitors, where the electrode pair works in the limited voltage ranges based on an open circuit voltage of approximately 3.0 V, corresponding to the two smaller CV curves in Fig. 3(d). This suggests that the electrochemical performance of 6-WP-HPPs can be improved when they are used over a wider voltage range. Therefore, an asymmetric electrode configuration using the 6-WP-HPPs in the full voltage range (1.0~4.5 V) rather than a conventional symmetric cell configuration is believed to be a better strategy to achieve higher energy storage. The cycling performance of the 6-WP-HPPs were also characterized by repetitive galvanostatic charge/discharge cycles at a current rate of 2 A g^−1^ [Fig. 3(e)]. The cycle number vs. capacitance retention plot reveals the highly reversible cycling performance of the 6-WP-HPPs even after more than 5,000 continuous charge/discharge cycles [Fig. 3(e)]. The initial capacity decreased slightly with increasing number of cycles; the initial capacity was reduced by only 20% after 5,000 cycles.

**Figure 4:**
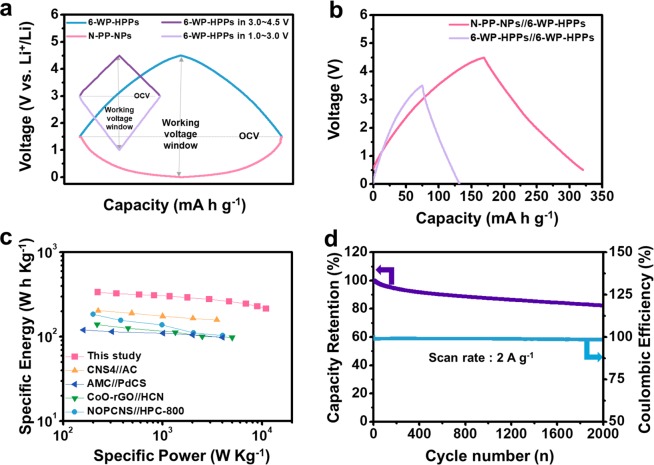
Electrochemical performance of LIHCs based on N-PN//6-WP-HPP. (**a**) Schematic diagram showing the charge/discharge voltage profiles of both symmetric and asymmetric full cells, (**b**) galvanostatic charge/discharge profiles of both symmetric and asymmetric full cells, (**c**) Ragone plots of several LIHCs, which include previously reported results and N-PN//6-WP-HPP cells, and (**d**) cycling performance of N-PN//6-WP-HPP cells at a current rate of 2 A g^−1^ over 2,000 cycles.

The 6-WP-HPPs-based full cell devices were assembled as both symmetric and asymmetric configurations to compare their electrochemical performance. The asymmetric cell was prepared using the previously reported pyropolymer anode (N-PN) after pre-cycling for 10 cycles^30^. Fig. S5 shows the electrochemical performance of the N-PN anode in an anodic voltage section for Li ion storage. The N-PN anode shows the high reversible capacity of >500 mA h g^−1^, great rate capability by 10 A g^−1^, and stable cycling behavior [Fig. S10]. Through the pre-cycling process, the poor Coulombic efficiency of 1^st^ cycle on both anode and cathode can be improved [Fig. S11] because the initial side reaction forming solid-electrolyte-interface layer occur in the pre-cycling process, and the voltages of both the anode and cathode were tuned to 1.5 V vs. Li^+^/Li as the onset potential [Fig. 4(a)]. From the onset potential, the 6-WP-HP cathode and N-PN anode were operated in voltage ranges of 1.5~4.5 V and 1.5~0.01 V, respectively, wherein the operating voltage window of the asymmetric full cell is extended to 4.5 V. In contrast, the symmetric full-cell based on 6-WP-HPPs//6-WP-HPPs have a maximum operating voltage window of ~3.5 V (cathode: 3.0~4.5 V and anode: 3.0~1.0 V vs. Li^+^/Li). Owing to the voltage-dependent charge storage behaviors, the 6-WP-HPPs reveal much smaller specific capacities in the narrow voltage range, corresponding to 3.0~4.5 V and 3.0~1.0 V for cathode and anode, respectively, leading to poor energy performance. The energy gap between the symmetric and asymmetric full cells can be confirmed by comparing their galvanostatic charge/discharge profiles [Fig. 4(b)]. The discharge capacity and average voltage of asymmetric full cells (N-PN//6-WP-HPP) were ~152 mA h g^−1^ and 2.23 V, respectively, wherein their energy density was calculated to be ~340 Wh kg^−1^. The energy density of the asymmetric cell was approximately four times higher than that of the symmetric cells. In addition, the power densities of the asymmetric cells were significantly higher than those of previously reported LIHCs at the same energy densities, which can be confirmed in the Ragone plots [Fig. 4(c)]^20,52–54^. A specific power density of ~1,200 and ~11,000 W kg^−1^ was achieved at ~300 and ~215 Wh kg^−1^, respectively, highlighting their superb power capabilities. Moreover, exceptionally high energy and power capabilities were maintained during a long-term cycling process of more than 2,000 repetitive cycles with a Coulombic efficiency of approximately 100% [Fig. 4(d)]. After 2,000 cycles, approximately 82% of the initial capacity was maintained, highlighting the good cycling stability of the asymmetric full cells. Therefore, the pseudocapacitive electrode pair based on a high-performance 6-WP-HPP cathode and a reported pseudocapacitive counterpart anode provides LIHCs with well-balanced energy/power characteristics and good cycling stability, realizing significantly high electrochemical performance.

## Conclusion

Waste natural polymer-derived pyropolymers, WP-HPPs, with a hierarchically nanoporous structure, high specific surface area, and numerous chalcogen functional groups, were fabricated using a simple carbonization and activation process. Systematic pore-size engineering of the WP-HPPs confirmed that a few nanometer-scale pores of approximately 3 nm play a key role in a pseudocapacitive charge storage because the open nanopores can transport solvated Li-ions easily into the inside of complex carbon structures and a large specific surface area can be provided by an effective active surface for charge storage. In a Li ion-based organic electrolyte, WP-HPPs delivered a high specific capacity of ~412 mA h g^−1^, which was well maintained at higher current rates (10 A g^−1^) and during repetitive cycling for more than 2,000 cycles. As a result, the significantly improved electrochemical performance of WP-HPPs, which surpasses those of conventional non-faradic carbon-based cathode materials, results from the well-balanced energy and power performance of the pseudocapacitive faradic anode. Furthermore, high specific energy and power of ~340 Wh kg^−1^ and ~11,000 W kg^−1^, respectively, can be achieved in non-aqueous asymmetric LIHCs. Moreover, the LIHCs based on WP-HPPs good cycling stability after more than 2,000 cycles.

## Supplementary information

Supplementary Information.
