# Supplementary material for: Hierarchically Nanoporous Pyropolymers Derived from Waste Pinecone as a Pseudocapacitive Electrode for Lithium Ion Hybrid Capacitors
Source: Sci Rep. 2020 Apr 2;10:5817. doi: 10.1038/s41598-020-62459-0 (PMC7118167; doi:10.1038/s41598-020-62459-0)
Supplement: Supplementary file 1 — Supplementary Information. [file 41598_2020_62459_MOESM1_ESM.docx]

**Supporting Information**

Hierarchically Nanoporous Pyropolymers Derived from Waste Pinecone as a Pseudocapacitive Electrode for Lithium Ion Hybrid Capacitors

*Jong Chan Hyun,^1,2^ Jin Hwan Kwak,^1^ Sang Moon Lee,^3^ Jaewon Choi,^4^ Kyu-Tae Lee^5^ and Young Soo Yun^1,2,*^*

*^1^Department of Chemical Engineering, Kangwon National University, Samcheok 25913 (South Korea)*

*^2^KU-KIST Graduate School of Converging Science and Technology, Korea University, 145 Anam-ro, Seongbuk-gu, Seoul, 02841 Republic of Korea*

*^3^Research Center for Materials Analysis, Korea Basic Science Institute(KBSI), 169-148 Gwahak-ro, Yuseong-gu, Daejeon 34133, Korea*

*^4^Department of Chemistry and Research Institute of Natural Sciences, Gyeongsang National University, Jinju 52828, Korea*

*^5^Department of Physics, Inha University, Incheon 22212, South Korea*

^*^Corresponding author. E-mail: [c-ysyun@korea.ac.kr](mailto:c-ysyun@korea.ac.kr)

**Keywords**: hierarchically porous; pyropolymer; nanoporous carbon; cathode; hybrid capacitor; asymmetric capacitor

**Table S1**. Comparison for specific capacities of reported carbon-based electrode materials.

| Sample | Surface area | Current density | Specific capacity | Potential range | Ref |
| --- | --- | --- | --- | --- | --- |
| Commercial AC | 1350 m^2^ g^-1^ | 0.1 A g^-1^ | 76.5 mAh g^-1^ | 2.0 ~ 4.5 V | [S1] |
| PHNCNB | 845 m^2^ g^-1^ | 0.1 A g^-1^ | 72 mAh g^-1^ | 2.5 ~ 4.5 V | [S2] |
| SLC | 2651 m^2^ g^-1^ | 0.1 A g^-1^ | 56 mAh g^-1^ | 3.0 ~ 4.5 V | [S3] |
| CPAC | 3011 m^2^ g^-1^ | 0.3 A g^-1^ | 135 mAh g^-1^ | 2.0 ~ 4.5 V | [S4] |
| MPC | 2674.8 m^2^ g^-1^ | 0.1 A g^-1^ | 59.6 mAh g^-1^ | 2.5 ~ 4.5 V | [S5] |
| PAC900 | 2167 m^2^ g^-1^ | 0.3 A g^-1^ | 115 mAh g^-1^ | 2.5 ~ 4.5 V | [S6] |
| eAC | 3250 m^2^ g^-1^ | 0.4 A g^-1^ | 128 mAh g^-1^ | 2.0 ~ 4.5 V | [S7] |
| PJ-AC | 2448 m^2^ g^-1^ | 0.1 A g^-1^ | 98 mAh g^-1^ | 2.0 ~ 4.0 V | [S8] |

[S1] Hu, Z. et al. Self-assembled binary organic granules with multiple lithium uptake mechanisms toward high-energy flexible lithium-ion hybrid supercapacitor. *Adv Energy Mater*. **8**, 1802273 (2018).

[S2] Liang, Tian. et al. A high-power lithium-ion hybrid capacitor based on a hollow N-doped carbon nanobox anode and its porous analogue cathode. *Nanoscale* **11**, 20715-220724 (2019).

[S3] Zhang, H. et al. Lithium ion capacitor with identical carbon electrodes yields 6 s charging and 100000 cycles stability with 1% capacity fade. *ACS Sustainable Chem. Eng.* **7**, 2867-2877 (2019).

[S4] Lu, Q. et al. Porous activated carbon derived from chinese-chive for high energy hybrid lithium-ion capacitor. *J. Power Sources* **398**, 128-136 (2018).

[S5] Zhu, G. et al. High energy density hybid lithium-ion capacitor enabled by Co_3_ZNC@N-doped carbon nanopolyhedra anode and microporous carbon cathode. *Energy Storage Mater*. **14**, 246-252 (2018).

[S6] Sun, F. et al. A high poerformace lithium ion capacitor achieved by the integration of a Sn-C anode and a biomass-derived microporous activated carbon cathode. *Sci. Rep*. **7**, 40990 (2017).

[S7] Li, B. et al. Activated carbon from biomass transfer for high-energy density lithium-ion supercapacitor. *Adv. Energy Mater*. **6**, 1600802 (2016).

[S8] Sennu, P., Aravindan, V., Ganesan, M., Lee, Y.-G., Lee, Y.-S. Biomass-derived electrode for next generation lithium-ion capacitors. *ChemSusChem* **9**, 849-854 (2016).


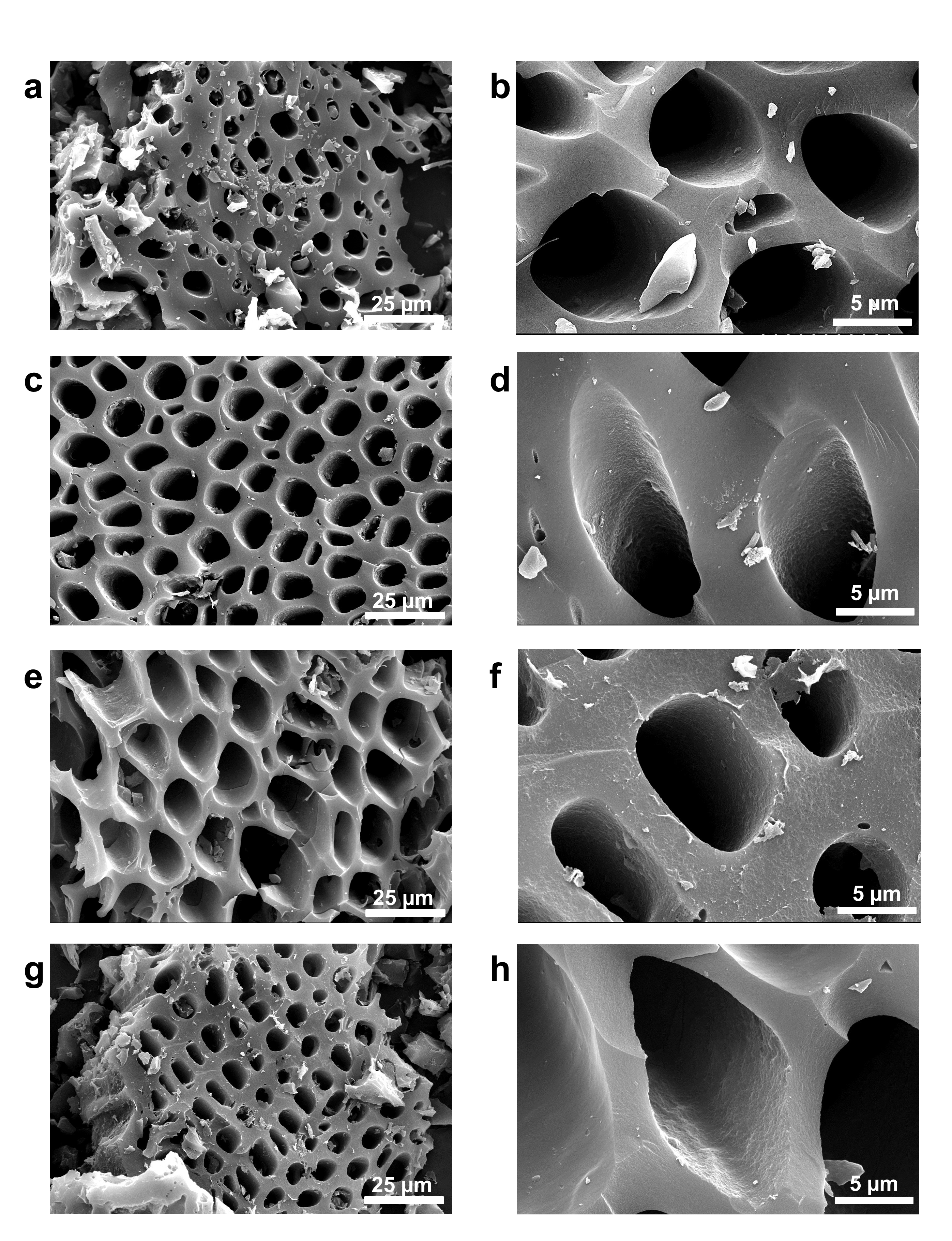


**Figure S1.** FE-SEM images of (a, b) pyropolymer precursors, (c, d) 2-WP-HPPs, (e, f) 4-WP-HPPs and (g, h) 8-WP-HPPs at different magnifications.


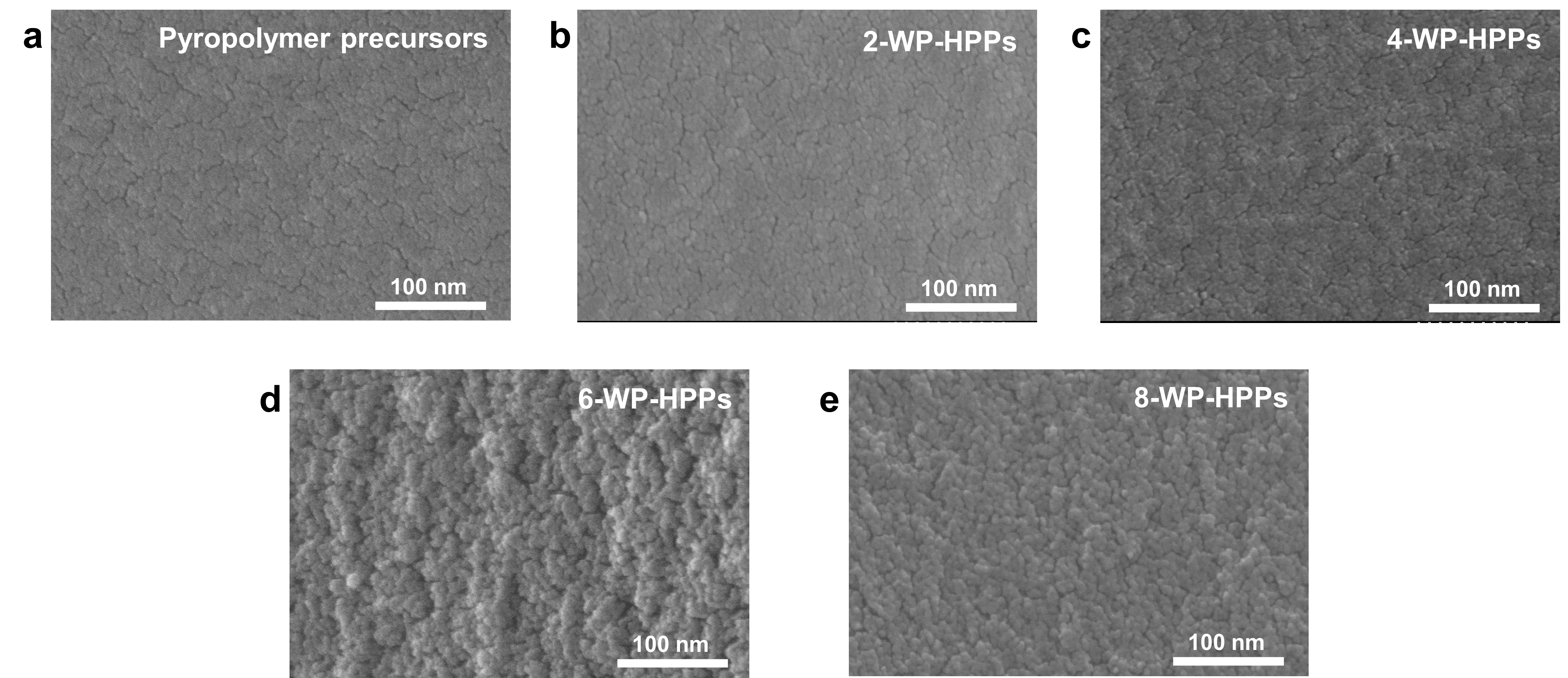


**Figure S2.** High resolution FE-SEM images of (a) pyropolymer precursors, (b) 2-WP-HPPs, (c) 4-WP-HPPs, (d) 6-WP-HPP and (e) 8-WP-HPPs.


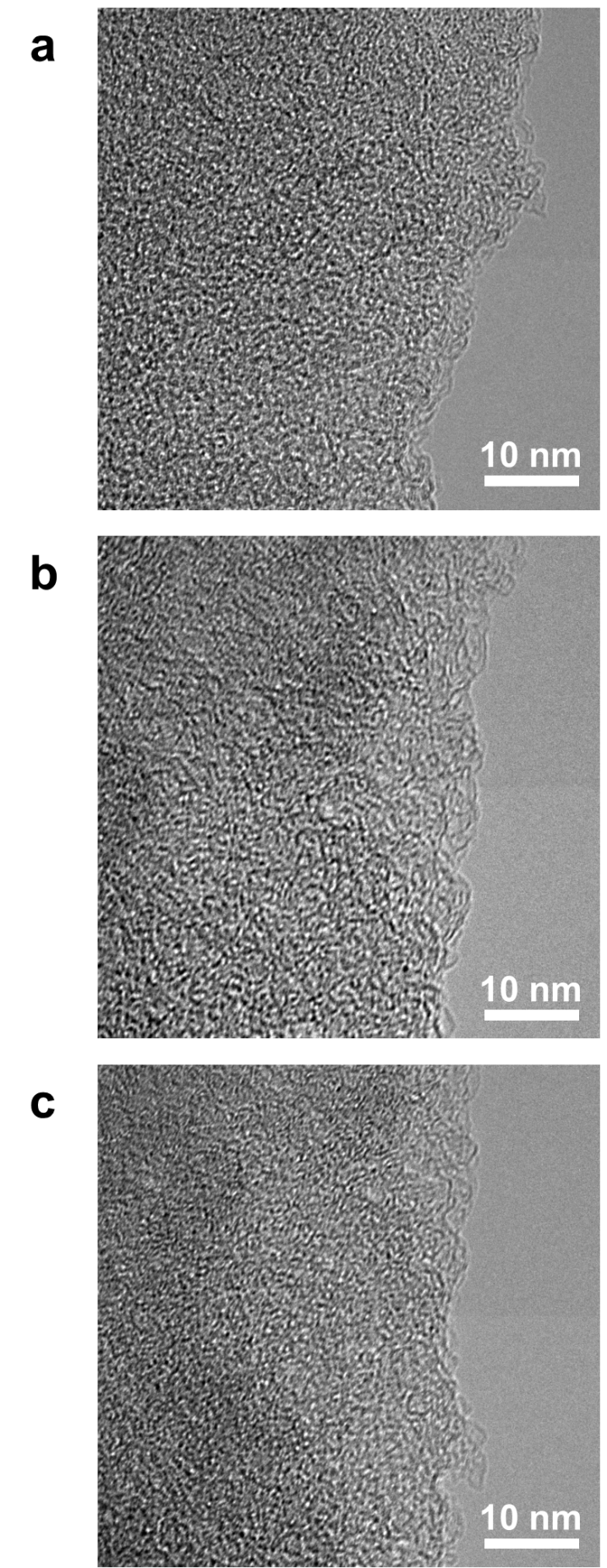


**Figure S3.** FE-TEM images of (a) 2-WP-HPPs, (b) 4-WP-HPPs and (c) 8-WP-HPPs.


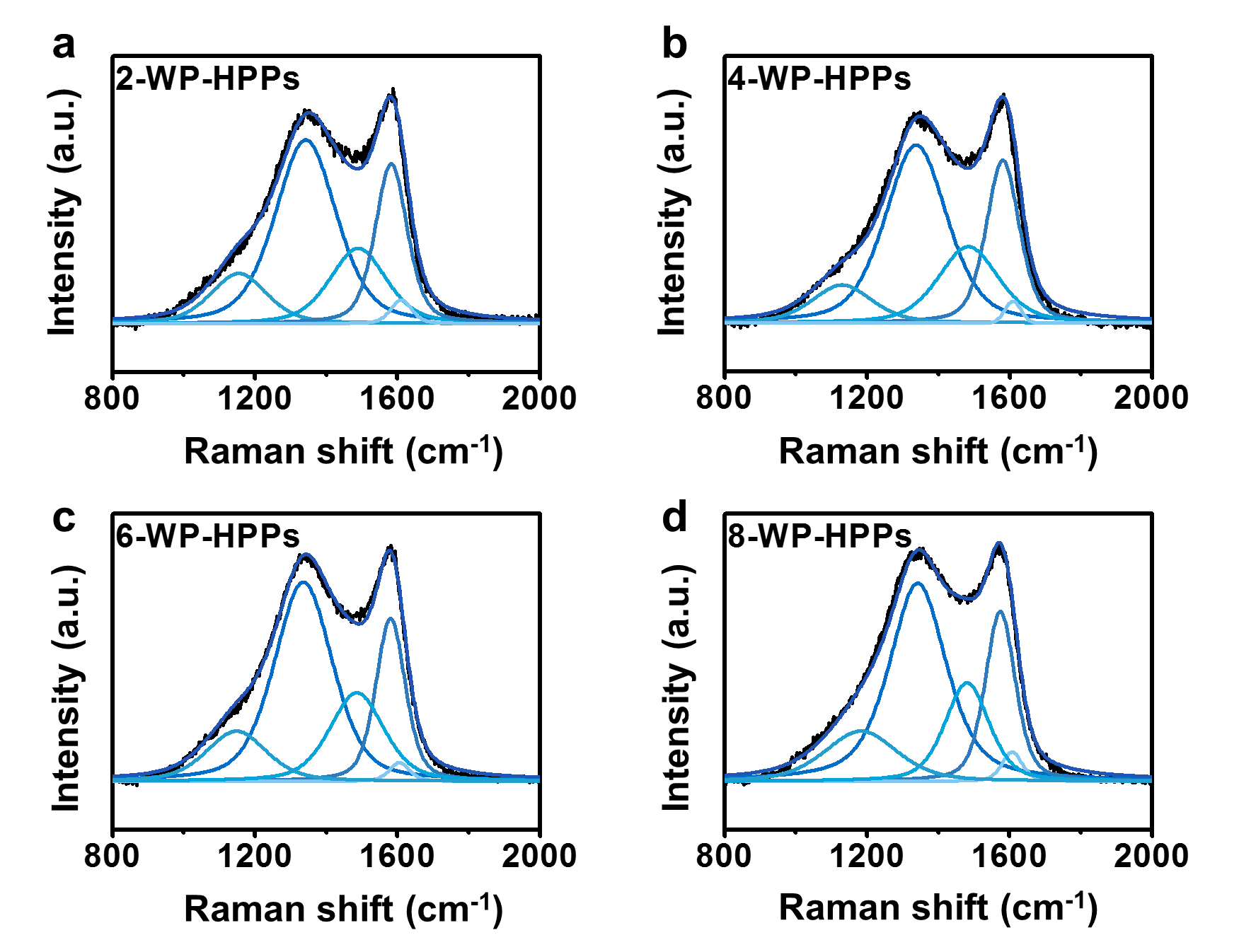


**Figure S4.** Deconvoluted Raman spectra of (a) 2-, (b) 4-, (c) 6- and (d) 8-WP-HPP samples.


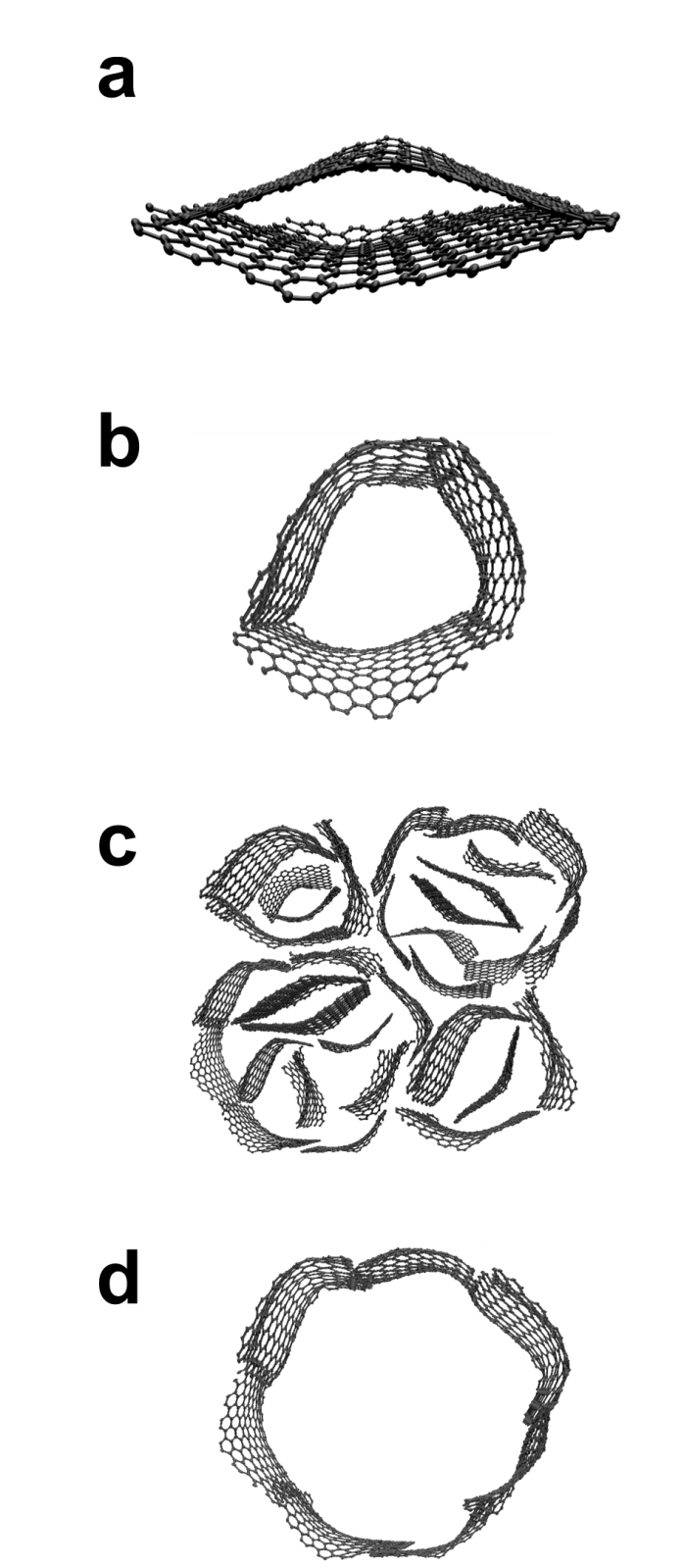


**Figure S5.** Schematic image showing different pore structures formed by asymmetric stacking of defective carbon BSUs: (a) ultra-micropore, (b) micropore, (c) ultra-micropore and micropore complex structure and (d) mesopore (~3 nm).

**
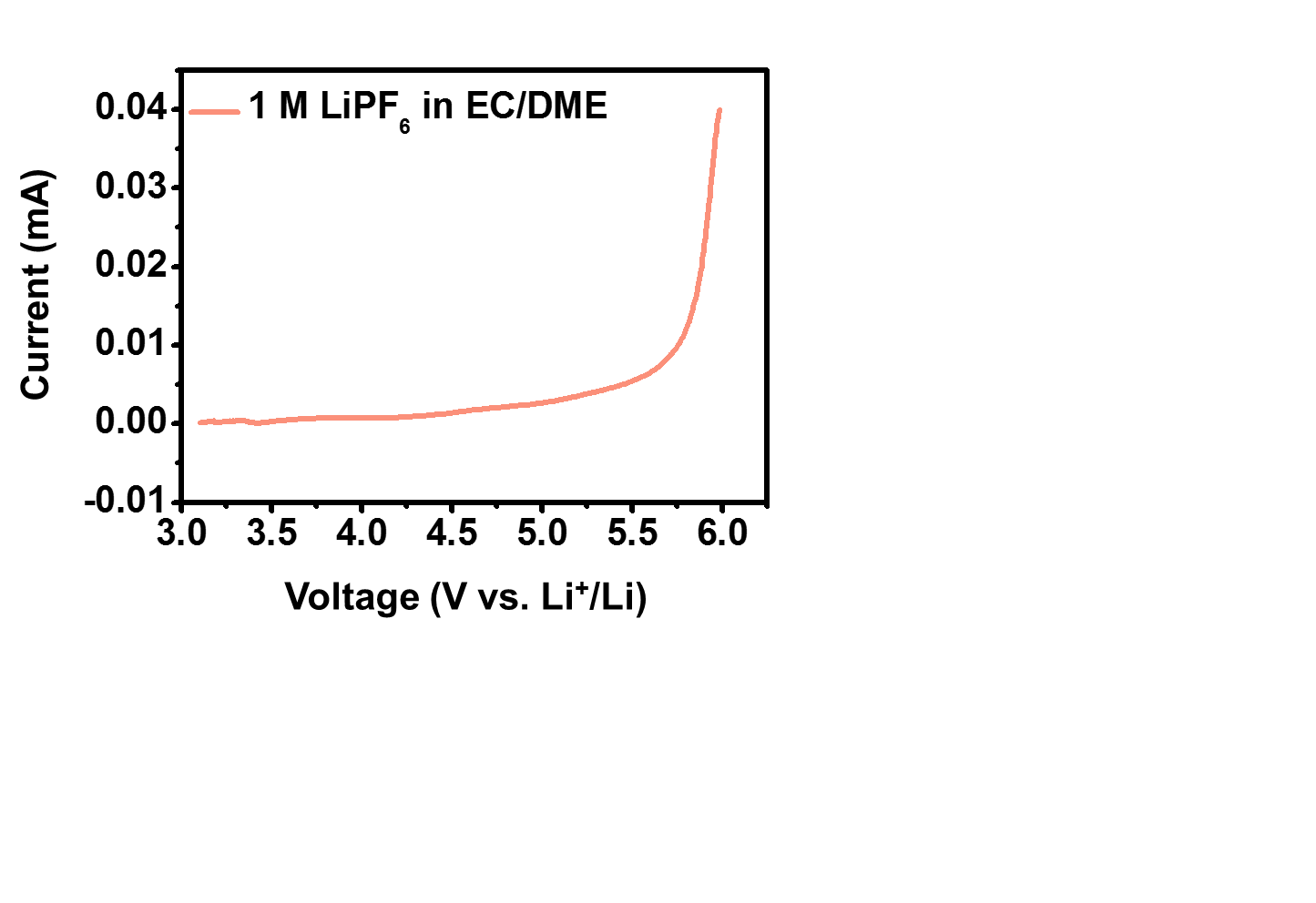
**

**Figure S6.** A linear sweep voltammetry curve characterized from open-circuit-voltage to 4.5 V at a scan rate of 1 mV s^-1^ for lithium metal//Al foil electrode in the EC/DMC mixture electrolyte.


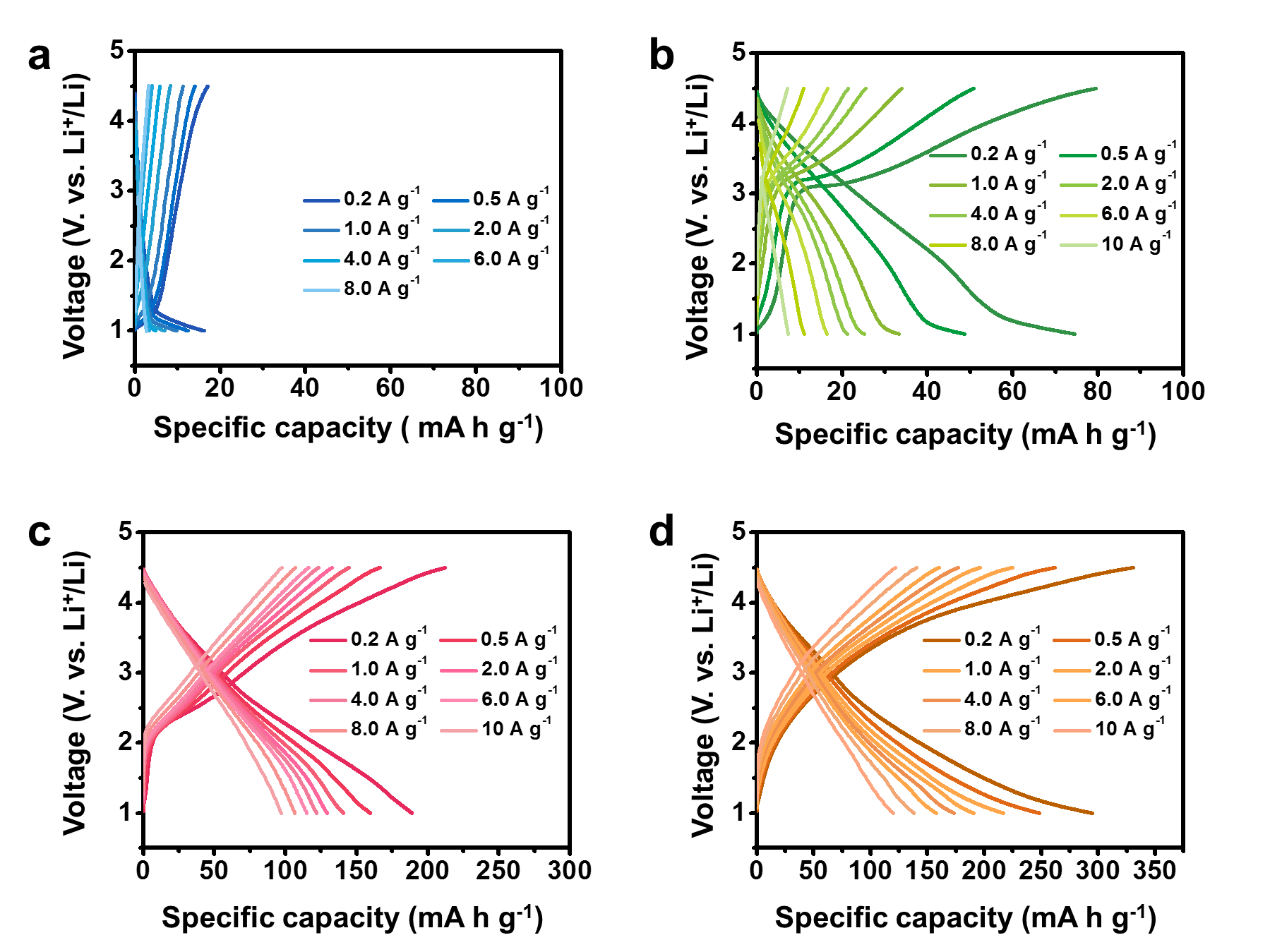


**Figure S7.** Galvanostatic charge/discharge profiles of (a) pyropolymer precursors and (b) 2-, (c) 4- and (d) 8-WP-HPPs in an electrolyte of 1 M LiPF_6_ dissolved in EC/DMC (1:1 v/v) over a voltage window of 1.0 ~ 4.5 V vs. Li^+^/Li.


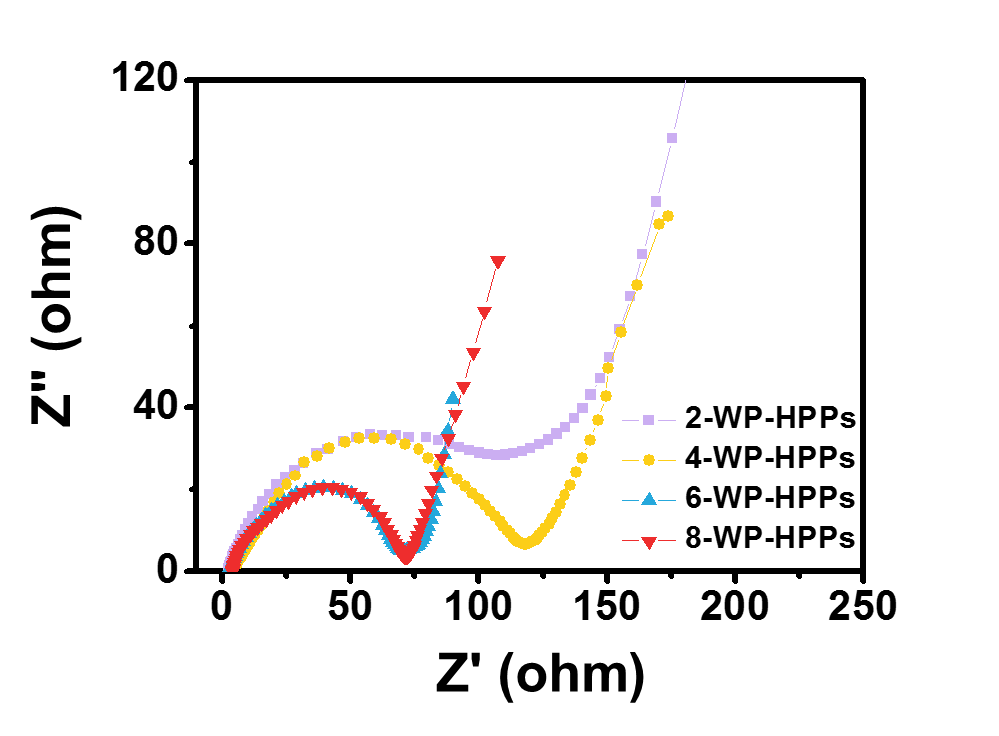


**Figure S8.** EIS profiles of WP-HPP samples characterized at room temperature in the frequency range of 0.1 MHz to 50 mHz.


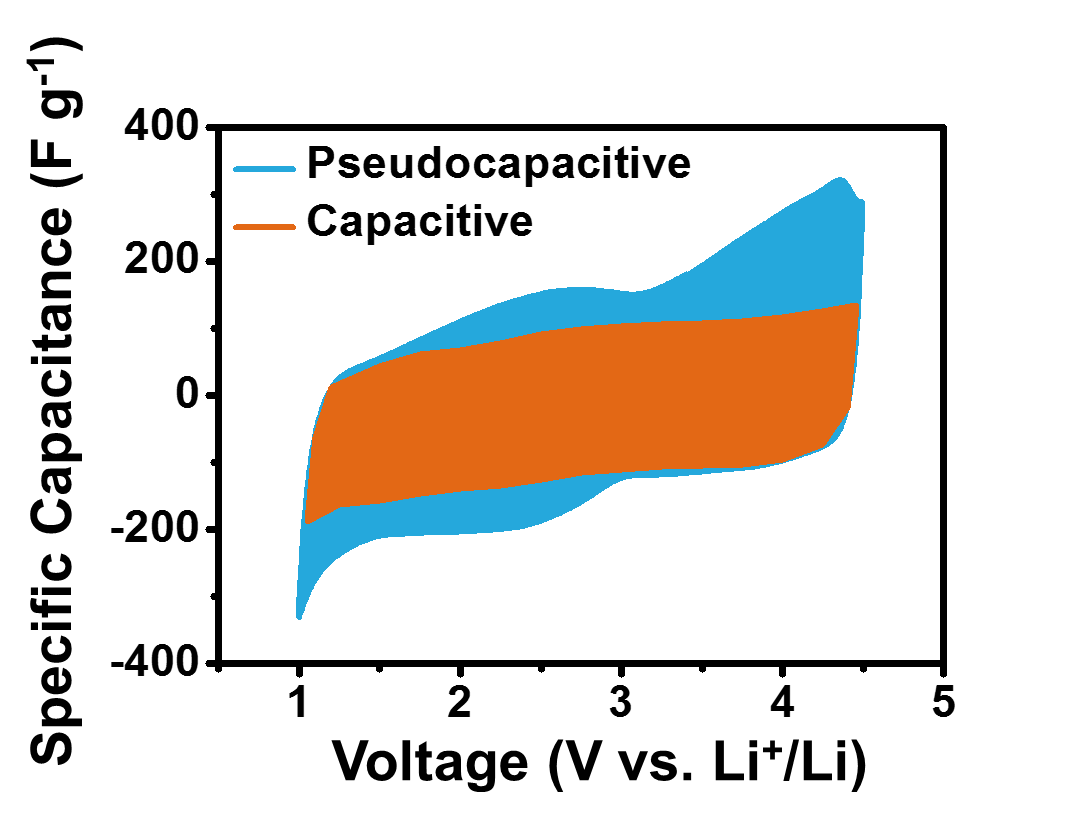


**Figure S9.** Quantitative ratio of the pseudocapacitive lithium ion storage capacitance in overall capacitance for 6-WP-HPPs.

**
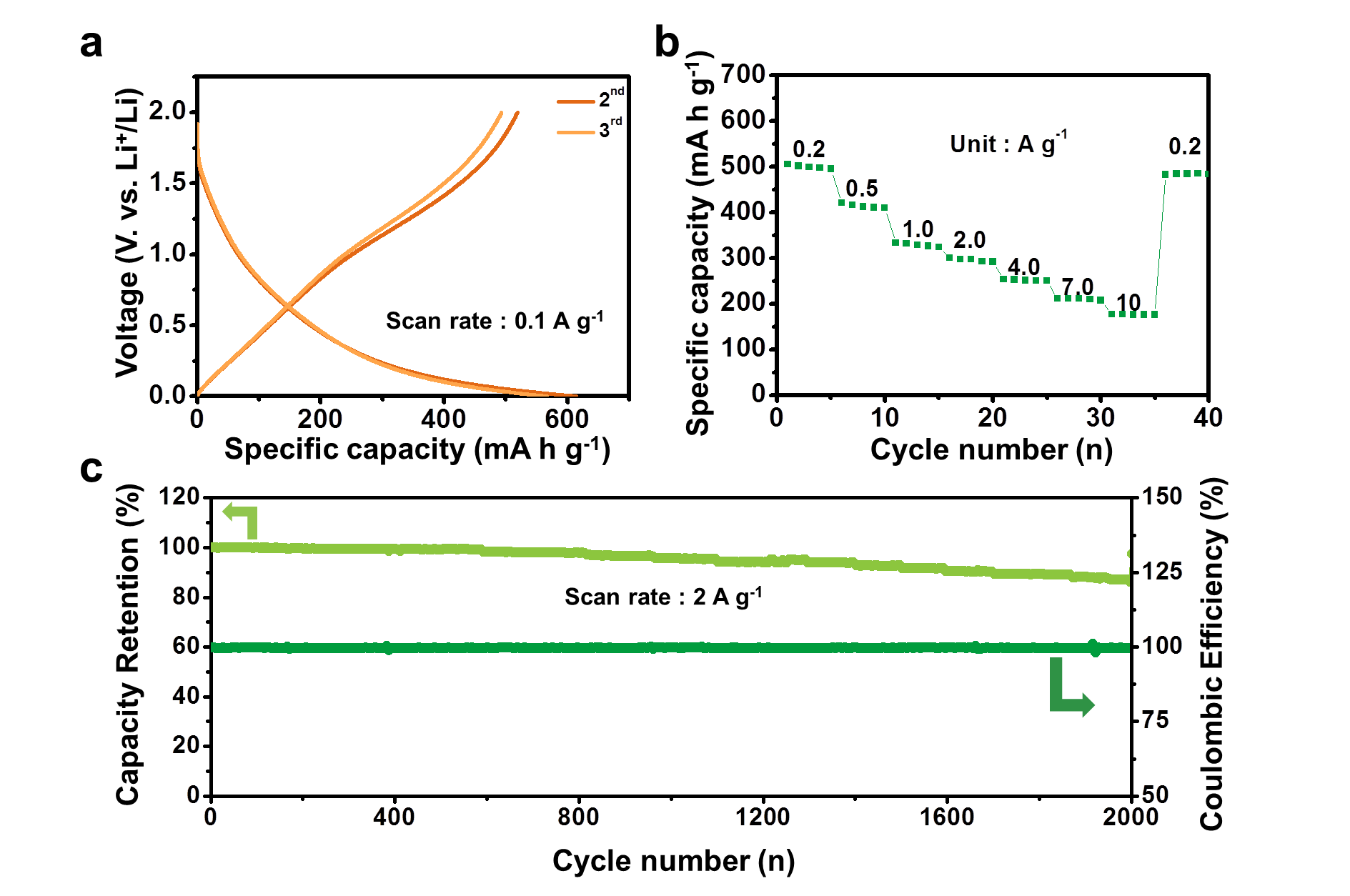
Figure S10.** Electrochemical performances of N-PN anode in an anode voltage section of 0.01 ~2.0 V vs. Li^+^/Li in an electrolyte of 1 M LiPF_6_ dissolved in EC/DMC (1:1 v/v). (a) Galvanostatic discharge/charge profiles at 0.1 A g^-1^, rate capabilities in different currernt rates from 2.0 to 10 A g^-1^ and (c) cycling performances at 2 A g^-1^ over 2,000 cycles.


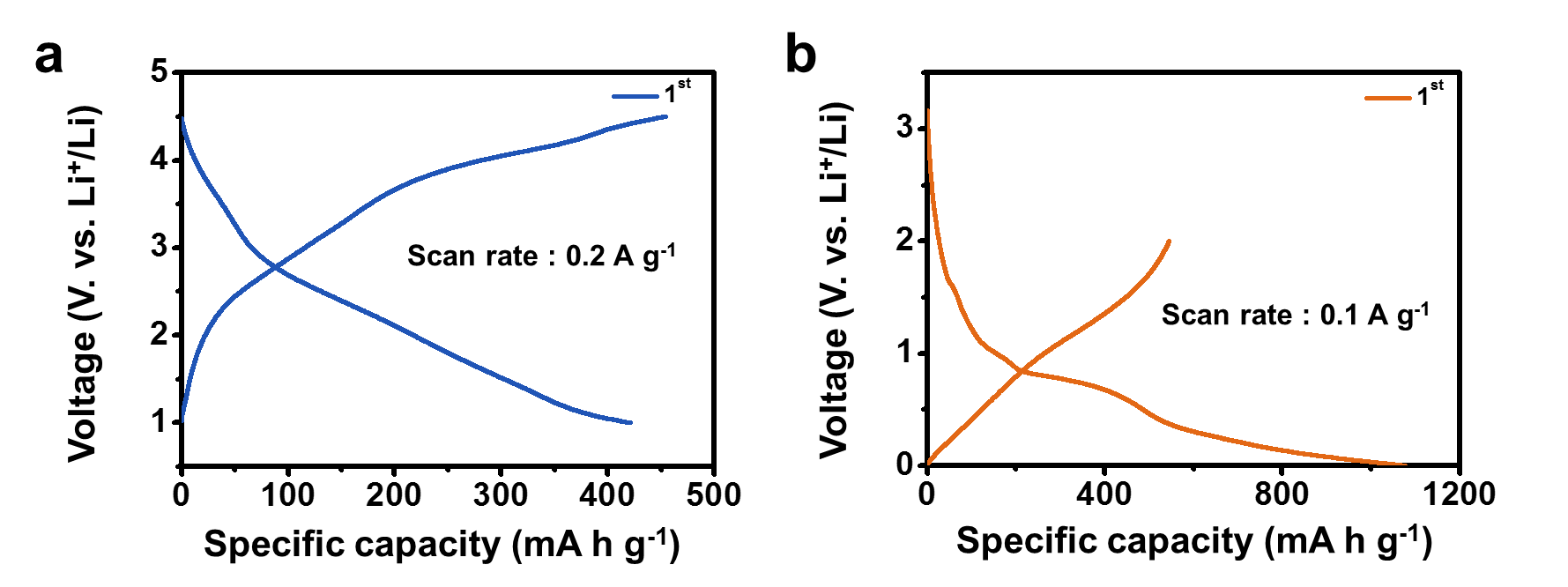


**Figure S11.** 1^st^ galvanostatic charge/discharge profiles of (a) 6-WP-HPP cathode and (b) N-PN anode characterized in the half-cell configuration.
